# Vascular endothelial protective effects of longan (*Dimocarpus longan Lour*.) extract and underlying molecular mechanisms

**DOI:** 10.3389/fphar.2026.1934125

**Published:** 2026-09-03

**Authors:** Wenliang Liu, Rui Sun, Yunlun Li, Yushuo Zhu, Lei Zhang, Feng Jiang

**Affiliations:** 1 First Clinical Medical College, Shandong University of Traditional Chinese Medicine, Jinan, China; 2 Affiliated Hospital of Shandong University of Traditional Chinese Medicine, Jinan, China; 3 Post-Doctoral Mobile Station of Shandong University of Traditional Chinese Medicine, Jinan, China

**Keywords:** cardiovascular disease, endothelial cells, longan, polyphenols, polysaccharides

## Abstract

Longan (*Dimocarpus longan Lour*.) is a traditional fruit with both nutritional and medicinal value, rich in various bioactive metabolites such as polysaccharides, phenolic acids, flavonoids, proanthocyanidins, triterpenoids, and adenosine. Following PRISMA 2020 guidelines, this narrative review systematically synthesizes the multi-target protective effects of longan’s bioactive metabolites on the vascular endothelial system, drawing on a systematic literature search of PubMed and Web of Science Core Collection (January 2015–July 2026) that employed Boolean combinations of keywords spanning plant nomenclature, vascular endothelium, and cardiovascular pharmacology, supplemented by manual screening of reference lists. Extensive studies using cellular and animal models have revealed that longan extracts and their metabolites effectively alleviate endothelial oxidative damage by directly scavenging free radicals. They downregulate the MAPK/NF-κB pathway, inhibit the expression of pro-inflammatory factors, and remodel the inflammatory microenvironment of the vascular wall. Regarding the regulation of vascular tone, longan metabolites exhibit bidirectional regulation of nitric oxide homeostasis. By inhibiting angiotensin-converting enzyme (ACE) activity, they reduce angiotensin II production and synergistically lower blood glucose, regulate blood lipids, and modulate immune balance. These findings suggest that longan and its bioactive metabolites hold significant potential for functional intervention in vascular diseases.

## 1 Introduction

Blood vessels form a tightly interconnected circulatory network that transports nutrients, oxygen, and metabolic waste, while also delivering cells, proteins, and signaling molecules of the immune system (Dimitrievska and Niklason, 2018). Cardiovascular disease (CVD) remains a leading cause of death worldwide, accounting for approximately one-third of global mortality (Soppert et al., 2020). Atherosclerosis, hypertension, and heart failure are among the major cardiovascular diseases. These conditions share a common pathological foundation, which is characterized by progressive structural and functional deterioration of the vascular wall that is driven by excessive oxidative stress, chronic low-grade inflammation, and dysregulated lipid metabolism (Stroope et al., 2024; Xu et al., 2025).

Within this pathological triad, the vascular endothelium serves as a critical first-line defense and a primary target for injury. The endothelium constitutively maintains vascular homeostasis by regulating barrier permeability, thrombogenicity, leukocyte trafficking, and vasomotor tone (Dimitrievska and Niklason, 2018). Endothelial dysfunction is increasingly recognized as a sentinel early event in CVD. This dysfunction emerges from a deleterious interplay among excessive reactive oxygen species (ROS) generation, diminished nitric oxide (NO) bioavailability, and sustained pro-inflammatory transcriptional activation. Mechanistically, oxidative stress uncouples endothelial nitric oxide synthase (eNOS), which shifts NO production toward superoxide anion generation and activates NF-κB-dependent inflammatory cascades that upregulate adhesion molecules, including VCAM-1 and ICAM-1, and recruit circulating monocytes (Heo et al., 2025; Mussbacher et al., 2022). This oxidative-inflammatory crosstalk also promotes endothelial-mesenchymal transition (EndMT) and extracellular matrix remodeling. Together, these processes establish a vicious cycle that precipitates atherosclerotic plaque formation and microvascular rarefaction (Heo et al., 2025). Given that treatment outcomes remain suboptimal, lifestyle modifications, particularly dietary management, are increasingly recognized as an important strategy to reduce vascular risk, maintain endothelial integrity, and prevent endothelial dysfunction (Elagizi et al., 2020; Nguyen et al., 2026; Patel et al., 2017). Dietary polyphenols have been shown to protect vascular endothelium through multi-targeted modulation of nitric oxide homeostasis, ACE activity, NF-κB signaling, and redox balance (Gai et al., 2023), providing a mechanistic rationale for investigating polyphenol-rich botanicals such as longan.

Longan (*Dimocarpus longan Lour*.) is an evergreen tree of the Sapindaceae. Its fruit (commonly known as longan) is not only a popular fruit throughout Asia but also a traditional medicine with a long history of use (Zeng et al., 2024; Zhao et al., 2025). Longan has occupied a distinguished position in traditional pharmacopoeias across South and Southeast Asia for centuries (Hong-In et al., 2021). In traditional Chinese medicine, longan is classified as a tonic botanical drug. It is warm in nature and sweet in taste. Clinically, it is used to treat symptoms such as palpitations, insomnia, forgetfulness, and neurasthenia (Park et al., 2010). In Bangladesh’s traditional herbal medicine system, longan is widely used to treat a variety of conditions, including gastrointestinal disorders, chickenpox, bone injuries, and neurological disorders, highlighting its unique status as a versatile herbal remedy (Paul et al., 2021). In traditional Southeast Asian medicine, longan is primarily used as a tonic and is widely employed to treat a wide range of health issues, including injuries, fever, snake bites, and menstrual irregularities (Zhao et al., 2025).

The multifaceted bioactivities of longan are derived from a complex array of constituents, including polysaccharides, phenolic acids (e.g., gallic acid, ellagic acid), flavonoids, hydrolyzable tannins (such as corilagin), proanthocyanidins, triterpenoids, and adenosine, distributed across its aril, pericarp, flowers, and seeds (Paul et al., 2021; Tang et al., 2019; Zhang et al., 2020; Zubairu et al., 2025). Longan polysaccharides (LP) and polyphenols, which include corilagin and gallic acid, significantly reduce oxidative stress, restore NO bioavailability, and inhibit NF-κB/MAPK-mediated inflammatory responses by activating the Nrf2/HO-1 and PI3K/Akt/eNOS signaling pathways, thereby protecting endothelial integrity. Furthermore, longan extract can bidirectionally regulate NO synthases, a process that entails promoting eNOS while inhibiting iNOS, enhance the activity of endogenous antioxidant enzymes, which comprise SOD and GSH-Px, and indirectly maintain vascular endothelial homeostasis through immune modulation, an example of which is the activation of macrophages via the TLR2/4 pathway (Kunworarath et al., 2016; Lan et al., 2021a; Wattanapitayakul et al., 2021; Zhang et al., 2025). These findings suggest that longan is a promising resource for the prevention and management of CVD associated with endothelial dysfunction. In addition, longan plays an irreplaceable role in treating diseases affecting other systems. The active metabolites in longan have also shown promise in improving Alzheimer’s disease, indicating its potential for neuroimmunomodulation (Zhang et al., 2025). The polyphenolic metabolites (such as gallic acid and ellagic acid) and polysaccharides found in longan have well-documented antitumor effects and constitute the primary active metabolites responsible for its antitumor properties (Zhang et al., 2020). Polysaccharides from longan pulp can alleviate intestinal damage both *in vivo* and *in vitro*, promote the expression of tight junction proteins, and exhibit prebiotic activity (Yue et al., 2022).

Beyond the above activities, a comparative analysis with its close relative lychee further highlights longan’s distinctive metabolite profile. Longan and lychee are closely related Sapindaceae species that share similar metabolite and pharmacological profiles. Both activate the eNOS/NO pathway to improve endothelial function, reinforcing a shared cardiovascular protective “family signature” within this family (Rangkadilok et al., 2005; Tang et al., 2019; Yue et al., 2022; Zhao et al., 2025). However, longan is distinguished by higher corilagin content with ACE-inhibitory potential and superior neuroprotection through adenosine, whereas lychee has a distinct polysaccharide and flavonoid profile (Nuchprapha et al., 2020; Tang et al., 2019).

Despite these encouraging developments, the existing literature on the endothelial protective effects of longan remains fragmented, and a dedicated synthesis that integrates the underlying molecular mechanisms is still lacking. Therefore, the purpose of this review is to comprehensively summarize the current evidence on the protective actions of *D. longan* extracts and their bioactive constituents on the vascular endothelium, with a particular emphasis on the signaling pathways involved and potential translational applications.

## 2 Search strategy

This narrative review was conducted following PRISMA 2020 guidelines. A systematic literature search was performed in PubMed and Web of Science Core Collection (January 2015–July 2026) using Boolean combinations of terms related to the plant and the vascular endothelium.

The following search strings were applied:

PubMed: (“Dimocarpus longan”[Mesh] OR “longan”[Title/Abstract] OR “Euphoria longana”[Title/Abstract]) AND (“endothelial cells”[Mesh] OR “cardiovascular diseases”[Mesh] OR “vasodilation”[Mesh] OR “nitric oxide”[Title/Abstract] OR “oxidative stress”[Title/Abstract] OR “inflammation”[Title/Abstract] OR “blood pressure”[Title/Abstract]) AND (“plant extracts”[Pharmacological Action] OR “polyphenols”[Pharmacological Action] OR “polysaccharides”[Pharmacological Action]).

Web of Science Core Collection: TS=(“Dimocarpus longan” OR “longan” OR “Euphoria longana”) AND TS=(“endothelial” OR “cardiovascular” OR “vascular” OR “NO” OR “nitric oxide” OR “antioxidant” OR “anti-inflammatory” OR “hypoglycemic” OR “ACE inhibitor”) AND DT=(Article OR Review) AND LA=(English).

The search was restricted to English-language original articles and reviews. A limited number of pre-2015 publications were included only if they provided (1) foundational mechanistic concepts of endothelial dysfunction, (2) seminal metabolite characterizations of longan, or (3) essential ethnopharmacological context. The search was supplemented by manual reference list screening.

Studies were included if they investigated longan-derived extracts or purified metabolites and reported at least one functional endothelial endpoint (NO production, cell viability, ROS, inflammatory cytokines, vasoreactivity, or endothelial biomarkers). Conference abstracts, case reports, editorials, and studies without quantitative data were excluded. Two reviewers independently screened titles/abstracts and full texts; discrepancies were resolved by a third reviewer.

To critically appraise the evidence, we adopted a hierarchical grading: human interventional studies as highest-tier, followed by animal models, with *in vitro* mechanistic studies assigned lower translational weight. For animal studies, we applied the SYRCLE Risk of Bias tool (randomization, blinding, allocation concealment); for *in vitro* studies, we assessed reproducibility, dose-response consistency, and use of controls. This quality appraisal informed the weighting of findings throughout the review. A detailed discussion of this methodological quality assessment is provided in Section 6.

**FIGURE 1:**
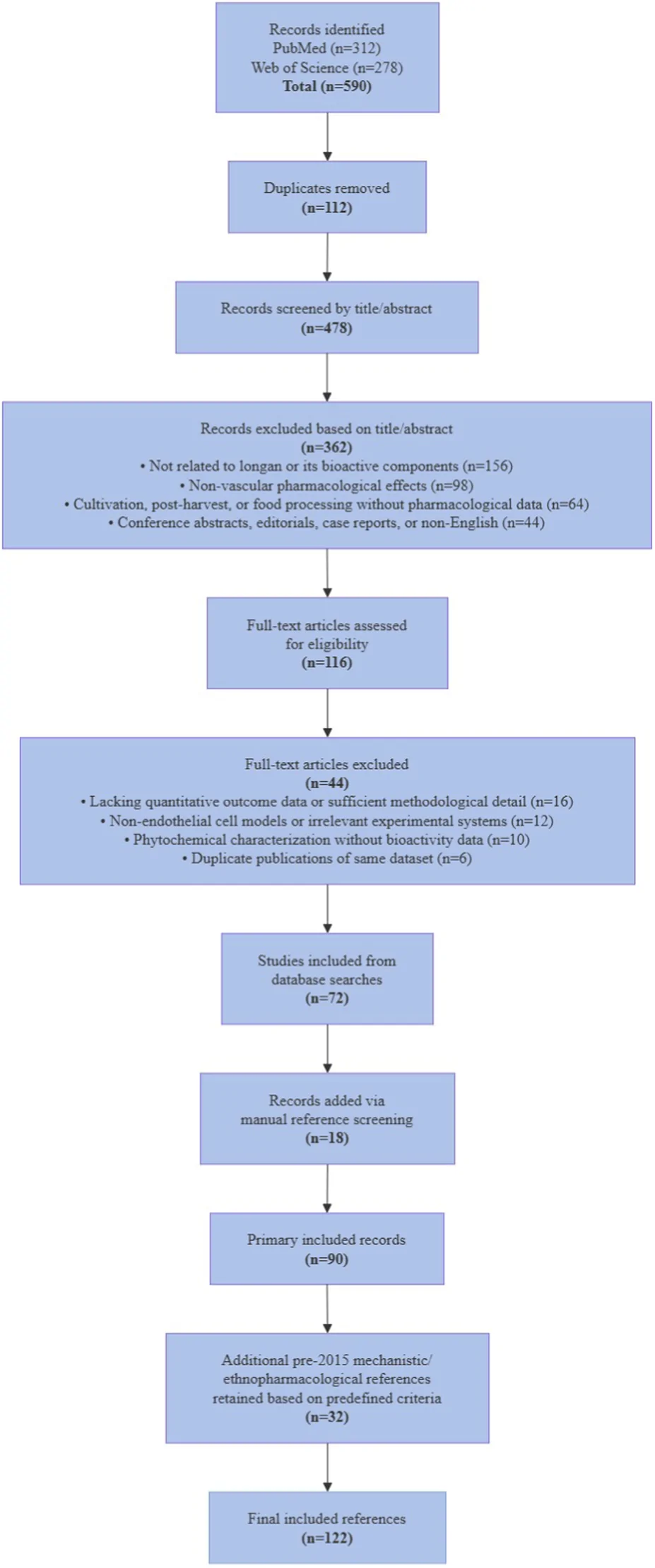
PRISMA 2020 flow diagram of the study selection process.

Details of the literature screening process, including the number of records identified, excluded, and finally included, are presented in the PRISMA 2020 flow diagram (Figure 1).

## 3 Bioactive metabolites in longan

### 3.1 An overview of the chemical composition of longan

Longan contains a wide variety of metabolites. To date, more than 378 metabolites have been isolated and identified from longan, including 12 sugars, 28 polyphenols, 234 volatile metabolites, 12 terpenes and steroids, 28 organic acids, 15 lipids, 11 nucleosides, 32 amino acids, and six other metabolites (Yi et al., 2011a). Key metabolites include polysaccharides and polyphenols (Zhao et al., 2025). A complete list of sugar compounds identified in longan is provided in Supplementary Table 1.

### 3.2 Longan polysaccharides

Research has shown that polysaccharides are macromolecules widely distributed in animals, plants, and microorganisms, and they play a significant role in cell proliferation, differentiation, and signal transduction (Xie et al., 2016). Natural polysaccharides possess a variety of pharmacological activities, such as antiviral, antitumor, and immunomodulatory activities (López-Legarda et al., 2020). Recent studies have identified a diverse array of bioactive polysaccharides from different parts of the longan fruit, with water-soluble polysaccharides being the most extensively investigated. These polysaccharides are composed of various monosaccharides and serve as the primary active macromolecules responsible for longan’s health benefits (Yue et al., 2022).

Among the identified metabolites, the glucose (Glc) is the most abundant monosaccharide in many longan polysaccharide fractions. Specifically, molecular weight fractions (≥50 kDa) are predominantly composed of glucose, forming α-(1→6)-linked glucans, which are abundant in both fresh and dried longan pulp. Polysaccharide fractions with high molecular weight (≥100 kDa) account for 28% of the total polysaccharides in fresh longan and 45% in dried longan (Lan et al., 2021c). Other studies have isolated specific polysaccharide fractions, such as FLP from fresh longan, which is mainly composed of mannose (Man) and glucose, and its counterpart DLP from dried longan (Gan et al., 2021). LP-F, a fermented longan polysaccharide, contains high levels of arabinose, galactose, rhamnose (Rha), and mannose, but low levels of neutral sugars and glucose (Huang et al., 2019b). Additionally, arabinose and mannose are other major monosaccharides found in various longan polysaccharide extracts (Huang et al., 2019a). Galacturonic acid (GalA) is also a key metabolite that contributes to the acidic nature of certain fractions, and the presence of uronic acids enhances the solubility and biological activity of these polysaccharides (Bai et al., 2017).

The most pivotal metabolites responsible for the pharmacological effects of LP are the heteropolysaccharides with high molecular weights and complex structures. Polysaccharides with high molecular weight (≥100 kDa) exhibit the most potent immunomodulatory activities by effectively activating macrophages and significantly promoting NO production and phagocytic activity. This is because these fractions can better engage with receptors such as TLR1/2 and TLR7/8 to activate the immune signaling pathways (Lan et al., 2021c). Studies have shown that a specific water-soluble polysaccharide called LP1 exhibits potent antitumor activity against SKOV3 and HO8910 tumor cells (with inhibition rates of 40% and 50%, respectively) and significantly enhances the proliferation of B cells and T cells (Meng et al., 2014). Similarly, LPS1, a water-soluble pure polysaccharide derived from longan pulp with a molecular weight of 1.08 × 10^5^ Da (Zhu et al., 2013), and LPD2 (Rong et al., 2019) have been shown to be immunologically active polysaccharides that promote cell proliferation and macrophage activity.

Critically, different extraction methods significantly influence the relative yield, composition, and bioactivity of LP. A systematic study comparing three methods—hot water extraction (LP-H), superfine grinding (LP-S), and superfine grinding-assisted enzymatic treatment (LP-SE)—revealed distinct outcomes. The LP-SE method exhibited superior performance: it significantly increased the extraction yield, total sugar content, solubility, and the percentages of arabinose and mannose compared to the other two methods. Conversely, this method led to a decline in the apparent viscosity, particle size, molecular weight (Mw), and glucose percentage. Regarding prebiotic activity, LP-SE showed the strongest stimulation effect on the proliferation of probiotics among the three tested fractions (Huang, et al., 2019c). In another method, ultra-high-pressure-assisted enzymatic extraction (UPE) maximized polysaccharide yield to 8. 55%, the highest among all tested samples; the resulting polysaccharide (LP-UE) also exhibited the highest acetylcholinesterase inhibitory activity, indicating that it is superior to conventional extraction methods (Bai et al., 2017). Fermentation also alters the composition of polysaccharides; polysaccharides (LP-0, LP-6, etc.) extracted from longan pulp fermented for different durations exhibit varying compositions and activities (Huang et al., 2019c). These studies help lay the groundwork for the application of longan in the production of functional foods and pharmaceuticals. Detailed characterization of longan polysaccharides is presented in Supplementary Table 2.

### 3.3 Longan polyphenols

Polyphenols are chemical metabolites naturally found in vegetables, fruits, grains, and beverages. In the late 20th century, epidemiological studies and related meta-analyses strongly suggested that long-term consumption of a diet rich in plant polyphenols offers some protection against the development of cancer, CVD, diabetes, osteoporosis, and neurodegenerative diseases. It plays a particularly important protective role in preventing metabolic disorders and chronic diseases (Ganesan and Xu, 2017; Pandey and Rizvi, 2009).

Using Ultra Performance Liquid Chromatography-Tandem Mass Spectrometry (UPLC-MS/MS), researchers identified 12 polyphenolic metabolites in eight longan varieties, including three phenolic acids—quinic acid, gallic acid, and phthalic acid—as well as nine flavonoids, such as naringin, rutin, hesperidin, and methylehesperidin (Wang et al., 2020). In addition to the metabolites identified above, corilagin is also recognized as a major polyphenolic metabolite in various longan tissues (Tang et al., 2019). Studies have shown that the polyphenol content in longan varies significantly depending on the growing region, specific fruit parts, and cultivar (Lin et al., 2016). Using a high-throughput reverse-phase high-performance liquid chromatography method, the major polyphenolic metabolites in longan fruit were identified as gallic acid, corilagin (a quercetin-derived tannin), and ellagic acid. The content of these three polyphenolic metabolites varied significantly among different plant tissues and varieties. In terms of tissue distribution, the highest levels of gallic acid, corilagin, and ellagic acid were found in the seeds, while the lowest levels were found in the pulp. Among commercial varieties, the Biewkiew and Edor varieties had the highest levels of gallic acid and ellagic acid, while the Srichompoo variety had the highest level of corilagin (Nuchprapha et al., 2020). Beyond these three primary polyphenols, additional studies have identified and quantified four polyphenolic substances—gallic acid, ethyl gallate, corilagin, and ellagic acid—in longan pericarp and seeds. Among these, corilagin is the major polyphenolic metabolite in both pericarp and seed, with higher alkaloid content in seeds compared to pericarp (Tang et al., 2019). See Supplementary Table 3 for a full list of longan polyphenolic compounds.

Different extraction methods significantly influence the yield, total phenolic content, and bioactivity of polyphenolic metabolites from longan tissues. Hot water extraction of longan seeds is an efficient method for obtaining high phenolic content and strong antioxidant activity (Tang et al., 2019). Ultrasonic-assisted extraction (UAE) of longan seeds is a green and efficient technique that significantly increases extraction yield and phenolic content (Yang et al., 2011). Ultra-high-pressure-assisted extraction (UHPE) at 500 MPa (UHPE-500) has demonstrated significant advantages. This method obtained higher phenolic acid contents compared to other high-pressure processing methods and conventional extractions, also showing higher extraction yields with high phenolic contents, along with superior antioxidant and antityrosinase activities. High-pressure extraction (HPE) also outperformed conventional extraction (CE) and ultrasonic extraction (UE) methods, exhibiting higher extraction efficiency in terms of extraction yield, phenolic content, and antioxidant activity (Prasad et al., 2010). These findings provide critical guidance for the optimization of extraction protocols aimed at maximizing the recovery of specific polyphenolic metabolites from longan tissues for functional food and pharmaceutical applications.

Longan polyphenols can directly scavenge free radicals and enhance the body’s own antioxidant defense capabilities, thereby alleviating oxidative stress (Zubairu et al., 2025). This fundamental antioxidant action synergizes with targeted anti-inflammatory effects (Rong et al., 2019). This synergistic effect is primarily achieved through the regulation of Mitogen-Activated Protein Kinase (MAPK), PI3K/Akt, NF-κB, and MyD88/TRAF6 signaling cascades (Lan et al., 2021a; Wang et al., 2024). Together, these actions produce significant immunomodulatory activity and hepatoprotective effects. They also provide neuroprotective benefits, including enhanced memory and anti-anxiety effects (Lin et al., 2012; Liu et al., 2012; Park et al., 2010). These metabolites may serve as essential metabolites for their various therapeutic effects in longans. See Table 1.

**TABLE 1:** Major active metabolites of longan and their distribution.

Tissue site	Active metabolites	Composition characteristics	Primary pharmacological activity	References
Fruit pulp	Polysaccharides (LP), gallic acid, ellagic acid	Highest polysaccharide content, lowest polyphenol content	Immune modulation, intestinal protection, antioxidant	Zhao et al. (2025)
Seeds	Corilagin, gallic acid, ellagic acid, ACE-inhibiting peptides	Highest polyphenol content	ACE inhibition, blood sugar reduction, antioxidant effects	Nuchprapha et al. (2020)
fruit peel	Proanthocyanidins, corilagin, gallic acid, ellagic acid	Rich in proanthocyanidins and corilagin	Antioxidant, anti-inflammatory	Bai et al. (2019)
Flower	Flavonoids, polyphenols	Highest flavonoid content	Anti-inflammatory (strongest NO inhibition), lipid-regulating	Kunworarath et al. (2016)

### 3.4 Structure–activity and dose–effect relationships of bioactive metabolites

The bioactivities of longan-derived metabolites are strongly influenced by their structural traits, yet systematic correlations remain insufficiently explored. For polysaccharides, molecular weight serves as a dominant factor. High-molecular-weight fractions (≥100 kDa) elicit superior immunomodulatory responses, which are attributed to multivalent binding to Toll-like receptors and the formation of supramolecular lattices that facilitate receptor clustering and downstream signaling (Lan et al., 2021a; Lan et al., 2021c). Monosaccharide composition also modulates the effects. Polysaccharides with a high galactose-to-arabinose ratio, such as LPIIa, exhibit notable anti-inflammatory activity through the suppression of NF-κB-mediated cytokine production (Bai et al., 2020a). Glycosidic linkage patterns determine receptor selectivity. α-(1→6)-linked glucans predominantly activate TLR4, whereas β-(1→4)-linked residues preferentially engage TLR2 (Lan et al., 2021a). For phenolic constituents, the number and spatial arrangement of hydroxyl groups directly govern radical-scavenging capacity. Corilagin, which possesses multiple galloyl moieties with a dense hydroxyl array, shows stronger DPPH and hydroxyl radical inhibition than gallic acid, which has fewer hydroxyl groups (Bai et al., 2019). Collectively, these structure-activity relationships offer a rational basis for the targeted application of longan bioactive metabolites in vascular protection.

The dose-response relationships of longan metabolites are governed by distinct biological mechanisms. For polysaccharides, the concentration required to achieve receptor saturation determines the efficacy threshold. High-molecular-weight fractions exhibit pronounced dose-dependent immunomodulatory activity because their multivalent binding capacity reaches the threshold for effective receptor crosslinking only above a critical concentration; below this threshold, insufficient receptor engagement fails to trigger full downstream signaling (Lan et al., 2021c). For phenolic metabolites, the dose-response curve follows a biphasic pattern: moderate concentrations maximize protection through Nrf2 activation and radical scavenging, while excessive concentrations diminish efficacy through pro-oxidant effects (Xiao et al., 2017). Tissue-specific differences further explain why seed polyphenols achieve ACE inhibition at substantially lower concentrations than pulp polysaccharides require for immunomodulation (Lan et al., 2021c; Nuchprapha et al., 2020). These mechanistic principles explain why specific doses outperform others and provide a framework for rational dose optimization. See Table 2.

**TABLE 2:** Comparison of structure-activity and dose-response relationships for longan polysaccharides and polyphenols.

Comparison dimensions	Polysaccharides (≥100 kDa)	Polyphenols (gallic acid/corilagin/ellagic acid)	References
Key Structural Determinants	Molecular weight, monosaccharide composition, types of glycosidic bonds	Number of Hydroxyl Groups and Their Spatial Arrangement	Lan et al. (2021a), Bai et al. (2019)
Receptor/Target	TLR2/4,TLR1/2,TLR7/8	Directly scavenges free radicals, ACE, and iNOS	Lan et al. (2021a), Lan et al. (2021b), Tang et al. (2019), Bai et al. (2019)
Dose-Response Curve Types	Threshold Dependence (Requires Reaching the Receptor-Crosslinking Concentration)	Biphasic nature (antioxidant at low concentrations, pro-oxidant at high concentrations)	Lan et al. (2021c), Xiao et al. (2017)
Tissue specificity	Source of Fruit Pulp → Immune Modulation	Seed Source → ACE Inhibition	Lan t al. (2021b), Lan et al. (2021c), Nuchprapha et al. (2020)

### 3.5 Comparative analysis of tissue-specific extracts

Longan tissues exhibit distinct bioactive profiles that determine their therapeutic applications. The pulp is rich in polysaccharides, which confer immunomodulatory activity and protect the intestinal barrier through TLR2/4-mediated pathways (Bai et al., 2020b; Lan et al., 2021c). The seed contains high levels of polyphenols, including gallic acid and ellagic acid, and provides non-competitive ACE-inhibitory peptides. These metabolites confer the strongest ACE-inhibitory and α-amylase inhibitory activities among all tissues (Nuchprapha et al., 2020; He et al., 2021). The pericarp is abundant in proanthocyanidins and corilagin, which contribute to the highest antioxidant capacity (Bai et al., 2019). The flower possesses the highest flavonoid content and exhibits the most potent anti-inflammatory activity through suppression of NF-κB and MAPK signaling (Kunworarath et al., 2016). These functional differences guide tissue-specific applications: seed extracts for antihypertensive and hypoglycaemic uses, flower extracts for anti-inflammatory purposes, pulp polysaccharides for immune health, and pericarp for antioxidant formulations.

### 3.6 Comparative analysis with other polyphenol-rich natural products

Beyond this family-level comparison, the analysis extends to a wider range of polyphenol-containing natural products, including green tea catechins, resveratrol, curcumin and pomegranate ellagitannins. Mechanistically, longan parallels these polyphenols by activating the Nrf2/ARE pathway (Bai et al., 2019; Pullikotil et al., 2012), inhibiting NF-κB (Heo et al., 2025; Kunworarath et al., 2016), and promoting eNOS phosphorylation via PI3K/Akt (Förstermann and Sessa, 2012; Wattanapitayakul et al., 2021). However, longan offers distinct advantages: high corilagin content with ACE-inhibitory activity (Tang et al., 2019), two novel ACE-inhibitory peptides from seeds (Nuchprapha et al., 2020), immunomodulatory polysaccharides acting through TLR2/4-MyD88 (Lan et al., 2021a; Lan et al., 2021b; Wang et al., 2024), and bidirectional NO regulation (Kunworarath et al., 2016; Wattanapitayakul et al., 2021). These features, together with tissue-specific functional specialization, position longan as a uniquely versatile multi-targeted dietary candidate that integrates polyphenolic, polysaccharide, and peptide-mediated mechanisms within a single fruit.

### 3.7 Quality control and standardization: current status and challenges

Standardization remains a critical gap that limits the translational potential of longan-based research, as the lack of uniform quality control criteria compromises the comparability of findings across studies. Extraction methods substantially influence the physicochemical properties and bioactivities of longan polysaccharides and polyphenols, yielding fractions with distinct molecular weight distributions and biological activities (Bai et al., 2017). Variability is further compounded by differences in cultivars, geographical origins, and harvest seasons (Lin et al., 2016; Wang et al., 2020). Several analytical approaches have been proposed to address these challenges. Rutin has been suggested as a chemical marker for seed extracts (Nuchprapha et al., 2020), while ethyl gallate has been proposed for leaf extracts (Tang et al., 2019). Despite these efforts, durable limitations persist: no consensus markers have been established, preparation protocols remain inconsistent, and a bioassay-based potency standard for endothelial protection is unavailable. Future work must prioritize consensus markers and activity-based standardization.

## 4 The vascular protective effects of longan in cardiovascular diseases

**FIGURE 2:**
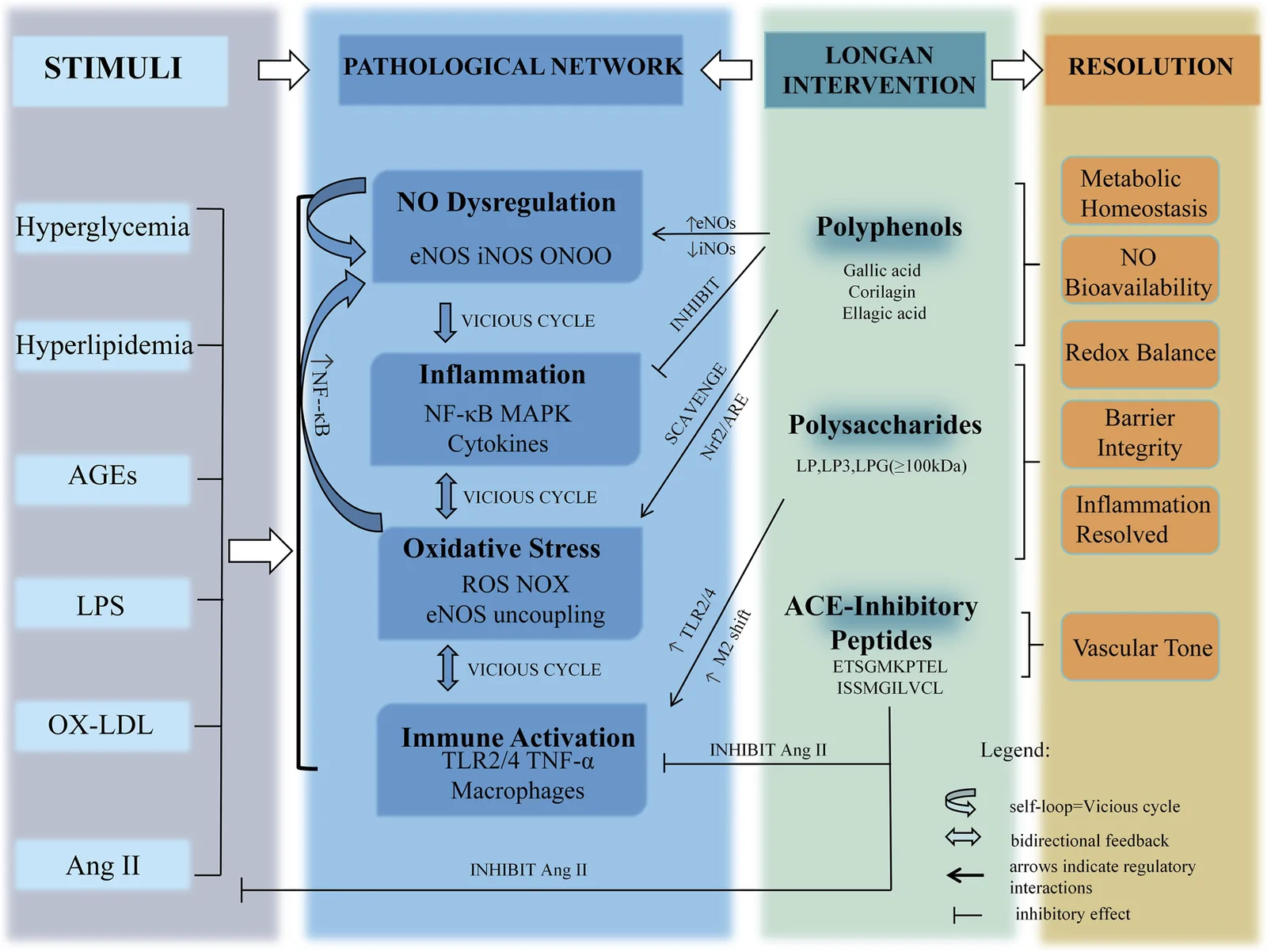
Multi-target mechanistic network of longan bioactive metabolites in endothelial protection. Upstream stimuli including hyperglycaemia, hyperlipidaemia, advanced glycation end products (AGEs), lipopolysaccharide (LPS), oxidised low density lipoprotein (ox LDL), and angiotensin II (Ang II) activate a pathological network comprising four interconnected modules. These are nitric oxide (NO) dysregulation involving endothelial NO synthase (eNOS) uncoupling, inducible NO synthase (iNOS), and peroxynitrite (ONOO); inflammation driven by nuclear factor kappa B (NF κB) and Mitogen-Activated Protein Kinase (MAPK); oxidative stress featuring reactive oxygen species (ROS) and NADPH oxidase (NOX); and immune activation via Toll like receptor 2/4 (TLR2/4) and tumour necrosis factor alpha (TNF α). Bidirectional feedback and vicious cycles link these modules. Longan polyphenols promote eNOS phosphorylation and inhibit iNOS through NF κB suppression. They also scavenge free radicals and activate nuclear factor erythroid 2 related factor 2/antioxidant response element (Nrf2/ARE). Longan polysaccharides modulate immune responses through TLR2/4 and promote macrophage polarisation toward an anti inflammatory phenotype. ACE inhibitory peptides reduce Ang II generation. These convergent actions restore NO bioavailability, resolve inflammation, maintain redox balance, preserve barrier integrity, and improve vascular tone and metabolic homeostasis. Abbreviations: ACE, angiotensin converting enzyme; AGEs, advanced glycation end products; Ang II, angiotensin II; eNOS, endothelial nitric oxide synthase; iNOS, inducible nitric oxide synthase; LPS, lipopolysaccharide; MAPK, mitogen activated protein kinase; NF κB, nuclear factor kappa B; NO, nitric oxide; NOX, NADPH oxidase; Nrf2/ARE, nuclear factor erythroid 2 related factor 2/antioxidant response element; ONOO, peroxynitrite; ox LDL, oxidised low density lipoprotein; ROS, reactive oxygen species; TLR2/4, Toll like receptor 2/4; TNF α, tumour necrosis factor alpha.

Endothelial protection by longan arises from a multi-target network that integrates antioxidant, anti-inflammatory, NO-modulating, immunomodulatory, hypoglycaemic, lipid-regulating, and ACE-inhibitory actions (Figure 2). These diverse mechanisms include direct scavenging of free radicals, chelation of iron ions, and activation of endogenous antioxidant enzymes such as SOD, CAT, and GSH-Px via the Nrf2/HO-1 pathway. Longan metabolites simultaneously inhibit MAPK and NF-κB signaling, which reduces the secretion of TNF-α, IL-6, IL-1β, and COX-2. A distinctive property of longan is its bidirectional regulation of NO homeostasis: polyphenolic metabolites stimulate protective eNOS phosphorylation through the PI3K/Akt pathway, while iNOS expression is suppressed via NF-κB inhibition, thereby preventing pathological NO overproduction and peroxynitrite formation. Immune modulation is achieved through TLR2/4-mediated macrophage activation, which restores the equilibrium between pro-inflammatory and anti-inflammatory mediators. The contributions of individual bioactive metabolites to these effects have been partially delineated. Corilagin contributes to antioxidant defense through its dense hydroxyl array and exhibits ACE-inhibitory activity that reduces angiotensin II generation (Bai et al., 2019; Tang et al., 2019). Gallic acid suppresses TNF-α, IL-6, and histamine secretion while promoting eNOS phosphorylation (Hong-In et al., 2021; Kim et al., 2006; Wattanapitayakul et al., 2021). Ellagic acid shares antioxidant and anti-inflammatory properties with gallic acid, but its rapid conversion to urolithins may confer a distinct pharmacokinetic profile (Tang et al., 2019). High-molecular-weight polysaccharides (≥100 kDa) mediate immunomodulatory effects through TLR2/4 pathways, indirectly protecting the endothelium from macrophage-driven inflammation (Lan et al., 2021c; Wang et al., 2024). Two novel ACE-inhibitory peptides derived from longan seeds provide an additional peptide-based mechanism for blood pressure regulation (Nuchprapha et al., 2020). These distinct contributions collectively explain how the diverse bioactive constituents of longan synergistically confer comprehensive endothelial protection. See Table 3.

**TABLE 3:** Multi-target cardiovascular protective effects of longan extracts.

Pharmacological activity	Key bioactive metabolites	Primary pathways/targets	Key endpoint measures	Level of evidence	References
Antioxidant	Polyphenols (corilagin), polysaccharides	Direct scavenging of DPPH/ABTS/•OH radicals; Fe^2+^ chelation; Nrf2/HO-1 activation; upregulation of SOD/CAT/GSH-Px	DPPH scavenging rate 71.8%; •OH inhibition rate 75.9%; Fe^2+^ chelation rate 36.4%; MDA↓; SOD/GSH-Px↑	*In vitro* + *in vivo (rodent)*	Bai et al. (2019)
Anti-inflammatory	Gallic acid, corilagin	Inhibition of MAPK (JNK/ERK/p38) and NF-κB phosphorylation; blockade of IκBα degradation; downregulation of AP-1/Akt	TNF-α↓; IL-6↓; IL-1β↓; COX-2↓; NO (iNOS-derived)↓	*In vitro* + *in vivo (rodent)*	Kunworarath et al. (2016)
Immunomodulation	High-MW polysaccharides (≥100 kDa)	TLR2/4→MyD88/TRAF6→PI3K/AKt; TLR1/2→Ca^2+^→MAPKs; CLR→Src/Raf1	Macrophage phagocytic activity↑70%–97%; NO (immune-derived)↑; NK cell cytotoxicity↑; Th1/Treg differentiation	*In vitro* + *in vivo (immunosuppressed model)*	Lan et al. (2021b), Lan et al. (2021c)
Hypoglycemic	Seed polyphenols	Non-competitive inhibition of α-amylase (H-bond/van der Waals binding); α-glucosidase inhibition	Postprandial blood glucose↓; fasting blood glucose↓; insulin sensitivity↑; α-amylase activity↓	*In vitro* + *in vivo (T2DM model)*	He et al. (2021)
Lipid regulation	Flower polyphenols, flavonoids	Pancreatic lipase inhibition (IC_50_ = 0.313 mg/mL); downregulation of SREBP-1c/FAS; upregulation of PPARα/LDLR	TG↓; TC↓; LDL-C↓; fecal fat excretion↑; atherosclerosis index↓	*In vitro* + *in vivo (high-calorie diet model)*	Yang et al. (2010)
Bidirectional NO regulation	Polyphenols (gallic acid, proanthocyanidins)	eNOS activation (promoting phosphorylation); iNOS inhibition (blocking NF-κB/AP-1 nuclear translocation)	Endothelial eNOS-derived NO↑; macrophage iNOS-derived NO↓ (flower extract IC_50_ = 128.2 μg/mL)	Primarily *in vitro* (EA.hy926 endothelial cells; RAW264.7 macrophages)	Kunworarath et al. (2016), Wattanapitayakul et al. (2021)
ACE inhibition	Seed-derived ACE inhibitory peptides (ETSGMKPTEL, ISSMGILVCL)	Non-competitive ACE inhibition (allosteric site binding; H-bond stabilization of enzyme-peptide complex)	ACE activity↓ (IC_50_ = 0.43 ± 0.011 mg protein/mL)	*In vitro (enzyme kinetics)*	Nuchprapha et al. (2020)

### 4.1 Antioxidant activity

Oxidative stress refers to an imbalance between the body’s oxidative and antioxidant systems; it is associated with the pathogenesis and prognosis of many vascular diseases (Zeng et al., 2024). Proper regulation of oxidative stress promotes angiogenesis and tissue repair (Huang and Nan, 2019). Free radicals are highly reactive molecules with unpaired electrons, but they have a dual effect: when the production of free radicals exceeds the capacity of the body’s antioxidant system, oxidative stress occurs (Hassan et al., 2024).

The clinical relevance of oxidative stress reduction lies in its direct impact on endothelial integrity. Excessive reactive oxygen species (ROS) damage the endothelium through three interrelated pathways. The first involves oxidation of tetrahydrobiopterin, which is an essential cofactor for endothelial nitric oxide synthase (eNOS). This oxidation causes eNOS uncoupling, a state in which the enzyme produces superoxide anions instead of nitric oxide (NO), thereby impairing vasodilation (Förstermann and Sessa, 2012; Janaszak-Jasiecka et al., 2023). A second mechanism is the activation of matrix metalloproteinases, which degrade the extracellular matrix and disrupt tight junctions, leading to increased vascular permeability (Hernandes et al., 2022). A third consequence is the triggering of mitochondrial apoptosis pathways, which reduces endothelial cell viability and compromises the structural integrity of the monolayer (Liang et al., 2022). Collectively, these pathways establish the causal link between oxidative stress and endothelial dysfunction. Longan extracts, which are rich in polyphenols and polysaccharides, can intervene at these three levels. Their ability to scavenge ROS directly and upregulate endogenous antioxidant enzymes enables them to counteract eNOS uncoupling, preserve barrier integrity, and limit endothelial cell loss, as detailed in the following subsections.

#### 4.1.1 Direct free radical scavenging and metal chelating activities of longan

Polysaccharides and polyphenols are the primary categories of bioactive metabolites in longan, and together they form the material basis for longan’s antioxidant activity (Zeng et al., 2024). Longan polyphenols and polysaccharides can effectively scavenge free radicals, providing an antioxidant effect to slow down cellular ageing (Zhao et al., 2025).

The molecular structure of longan polyphenols is rich in phenolic hydroxyl groups, which can efficiently scavenge 1,1-diphenyl-2- picrylhydrazyl radicals (DPPH) and 2,2′-azino-bis(3-ethylbenzothiazoline-6-sulfonic acid) (ABTS) radicals. Longan polyphenols exhibit excellent DPPH radical scavenging capacity and FRAP reducing power, demonstrating their outstanding *in vitro* direct radical scavenging activity (Wang et al., 2023). The polyphenols in longan exert antioxidant activity by reducing iron ions to directly neutralize free radicals, such as DPPH and ABTS, and reactive oxygen species. These polyphenolic metabolites protect cells from oxidative stress damage primarily by consuming oxygen and scavenging free radicals (Wang et al., 2020). The longan polyphenol corilagin exhibited the highest DPPH radical scavenging rate (71.8% ± 0.5%) and hydroxyl radical inhibition rate (75.9% ± 0.3%), in terms of Fe^2+^ chelating ability, protocatechuic acid exhibited the strongest chelating activity, with a chelation rate of 36.4% ± 0.7% (Bai et al., 2019). Polyphenol extract from longan by-products (LPPE) was evaluated using DPPH, ABTS, and FRAP assays. The results indicate that LPPE exhibits excellent antioxidant activity (Tan et al., 2023).

The polysaccharides in longan pulp exhibit good scavenging activity against DPPH radicals and hydroxyl radicals, and this activity is concentration-dependent (Yue et al., 2022). LP exhibited strong antioxidant activity in DPPH and superoxide anion radical scavenging assays, and this effect was dose-dependent (Jiang et al., 2009). LP extracted using ultrasonication exhibited excellent scavenging activity against hydroxyl radicals and DPPH radicals, demonstrating nearly complete scavenging efficacy (Zhong et al., 2010). In a study, longan crude polysaccharides (LP) were isolated and purified to yield longan polysaccharide LP-A. When the concentrations of LP and LP-A were 0.75 mg/mL and 0.5 mg/mL, respectively, the ABTS radical scavenging rate reached 99% (Guo et al., 2024). The purified acidic polysaccharide LP-A was isolated from LP; its ABTS radical scavenging rate reached 99% *in vitro*. In an *in vivo* model using *Caenorhabditis elegans*, it extended the average lifespan of the nematodes by 25.80% and increased their survival time by 69.57% under oxidative stress (Guo et al., 2024).

#### 4.1.2 Activation of endogenous antioxidant defense systems

Antioxidant enzymes help reduce the accumulation of harmful oxidative stress. Water extracts from fruit peels (WLP) significantly increase antioxidant enzyme activity in mice, including catalase (CAT), superoxide dismutase (SOD), and glutathione peroxidase (GSH-Px), which play a crucial role in antioxidant defense. LP significantly reduced malondialdehyde (MDA) levels in rats with cerebral ischemia-reperfusion injury and increased the activity of SOD, glutathione (GSH), and GSH-Px (Chen et al., 2011). Longan flower extract increased the activity of major antioxidant enzymes, including GPx and Superoxide dismutase (SOD), and elevated intracellular glutathione levels. These findings are consistent with previous studies on phenol-rich plant extracts, which protect cells from oxidative stress by directly scavenging free radicals and upregulating endogenous defense systems (Ma et al., 2025).

#### 4.1.3 Factors influencing antioxidant activity

At the same time, variations in the fruit concentration of longan can also affect the expression of its antioxidant activity (de Assis et al., 2009). There is a strong correlation between polyphenol content and antioxidant activity. The “Dingyuan” variety has the highest polyphenol content and exhibits significantly higher antioxidant activity than other varieties (Lin et al., 2022). In tests of the antioxidant capacity of phenolic metabolites in eight longan varieties, all exhibited strong iron-reducing antioxidant activity (Wang et al., 2020).

Longan Pulp Wine Polysaccharides (LWP), the primary active metabolite of longan pulp wine, were isolated from longan pulp wine using an ultrafiltration system into four fragments. The results showed that as molecular weight (Mw) increased, the scavenging activity of longan pulp wine and the four LP fractions against •DPPH gradually increased. Compared with longan pulp wine, LWP fragments of 10–30 kDa and <3 kDa exhibited significant antioxidant activity within a volume range of 0. 2–2. 0 mL; when the sample volume was 0. 2–1. 6 mL, the LWP fragment of 10–30 kDa showed the highest scavenging rate of DPPH (Liu et al., 2018).

Therefore, longan extract may exert its antioxidant effects through a dual mechanism: on the one hand, its abundant phenolic metabolites act as reducing agents, directly neutralizing and scavenging free radicals such as DPPH, ABTS, and hydroxyl radicals, while simultaneously chelating iron ions that catalyze oxidative reactions, thereby blocking the oxidative chain reaction at its source; on the other hand, it enhances the activity of key antioxidant enzymes such as SOD, CAT, GSH-Px, increasing GSH levels, and reducing the formation of the lipid peroxidation product MDA, thereby synergistically alleviating cell damage caused by oxidative stress.

### 4.2 Anti-inflammatory activity

Vascular damage caused by inflammatory responses plays a pivotalrole in the development of CVD (Fan and Watanabe, 2022). Inflammatory responses compromise vascular endothelial function by driving the release of pro-inflammatory cytokines and the inducible NO synthase-mediated overproduction of NO, which reacts with superoxide to generate peroxynitrite and thereby directly mediates oxidative endothelial injury central to CVD (Luecke et al., 2021; Vervaeke and Lamkanfi, 2025; Wautier and Wautier, 2023). NO exhibits concentration-dependent bidirectional regulation in inflammation (Piacenza et al., 2022).

Endothelial inflammation actively drives the initiation and progression of cardiovascular disease through multiple interconnected mechanisms. Under stimulation by pro-inflammatory cytokines such as TNF-α and IL-1β, the NF-κB pathway is activated in endothelial cells, which upregulates adhesion molecules including VCAM-1 and ICAM-1. These molecules facilitate monocyte adhesion to the endothelium, a critical early step in atherosclerotic plaque formation (Libby et al., 2011). In parallel, inflammatory mediators increase endothelial permeability by disrupting tight junction proteins, promoting subendothelial lipid deposition and foam cell formation (Hansson and Hermansson, 2011). Chronic inflammation also shifts the endothelium toward a pro-thrombotic state through tissue factor upregulation and thrombomodulin downregulation, increasing the risk of atherothrombotic events (Steffel et al., 2006). Furthermore, inflammatory signals promote endothelial-mesenchymal transition (EndMT), which contributes to vascular fibrosis and stiffening (Heo et al., 2025). Collectively, these mechanisms explain how endothelial inflammation serves as a central driver of vascular disease pathogenesis.

Interventions that suppress pro-inflammatory cytokine production and NF-κB activation can therefore counteract these pathogenic pathways at their source. The anti-inflammatory targets of various cardiovascular protective drugs lie in the inhibition of the TNF-α/NF-κB signaling pathway (Laveti et al., 2013). Numerous studies have confirmed that inflammatory responses in vascular endothelial cells are similarly driven by this pathway (Liu et al., 2020). Buckwheat flavonoids have recently been shown to suppress LPS-induced COX-2 signaling in RAW264.7 macrophages(López-Cánovas et al., 2026), corroborating the convergence of dietary flavonoids on the TLR4/NF-κB axis. Longan flower extract, however, achieves markedly superior potency (IC_50_ = 128.2 μg/mL for NO inhibition) (Kunworarath et al., 2016), underscoring its distinct advantage within this anti-inflammatory framework. Longan extract inhibits LPS-induced activation of the MAPK (JNK, ERK, p38) and NF-κB signaling pathways, blocking the degradation of IκBα and downregulating transcriptional regulatory nodes such as Activator Protein 1 (AP-1) and Akt, thereby inhibiting the expression of iNOS and COX-2, and consequently reducing the production of pro-inflammatory molecules such as TNF-α, IL-6, IL-1β, prostaglandin E2 (PGE_2_), and high concentrations of NO. In one study, a novel black D. longan pulp extract exhibited the strongest inhibitory effect on the secretion of inflammatory cytokines; the gallic acid it contains is believed to be the primary active metabolite responsible for its inhibitory activity against IL-6 and TNF-α (Hong-In et al., 2021). Gallic acid and ellagic acid in longan significantly inhibited NO production; gallic acid also inhibited histamine release from mast cells and the production of pro-inflammatory cytokines (Kim et al., 2006). Longan pulp polysaccharide LPIIa exerts its anti-inflammatory effects by inhibiting key proinflammatory factors such as TNF-α, IL-6, and COX-2, a process whose underlying molecular mechanism involves the suppression of NF-κB signaling pathway activation (Bai et al., 2020a). Longan seed extract, LSE, inhibited the production of NO, interleukin-1 beta, IL-6 and COX-2 induced by lipopolysaccharide or carrageenan. LSE suppressed the lipopolysaccharide-activated c-Jun N-terminal kinase, or JNK, the extracellular signal-regulated kinases, or ERKs, and the p38 MAP kinase signaling pathway (Lee et al., 2016). Studies have shown that longan flower extract exhibits the strongest inhibitory effect on the production of NO, followed by longan seed and pulp extracts. These extracts also suppress the degradation of IκBα induced by lipopolysaccharide, or Lipopolysaccharide (LPS), and the activation of NF-κB, activator protein-1, or AP-1, Akt, and mitogen-activated protein kinases, or MAPKs (Kunworarath et al., 2016). Furthermore, the aqueous extract of longan pericarp, designated as WLP, exhibited a concentration-dependent inhibitory effect on carrageenan-induced paw edema in mice, confirming that the *in vitro* anti-inflammatory activity can be translated into effective anti-inflammatory efficacy *in vivo* at the tissue level (Huang et al., 2012).

### 4.3 Regulation of immune system activity

Macrophage dysregulation constitutes a core immunoregulatory mechanism that underlies endothelial injury in CVD. Macrophages, which serve as key immune effector cells within the cardiovascular system, regulate the inflammatory microenvironment through a dynamic equilibrium in which they secrete proinflammatory cytokines such as TNF-α and IL-1β to enhance immune responsiveness, and simultaneously participate in inflammation resolution and tissue repair by releasing anti-inflammatory factors (Zhu et al., 2025). Longan has demonstrated anti-inflammatory activity by inhibiting pro-inflammatory factors. Effective immune regulation can restore the balance between pro-inflammatory and anti-inflammatory mediators, inhibit pathological endothelial activation, and maintain vascular homeostasis (Kim et al., 2025; Naderi-Meshkin and Setyaningsih, 2024).

Modulating the activity of immune cells is the cornerstone of immune regulation, as it initiates and enhances immune responses by activating innate and adaptive immune cells (Liu et al., 2025). Longan polysaccharide–protein complexes, such as LP1, LP2, and LP3, significantly stimulate splenic lymphocyte proliferation, enhance macrophage phagocytic activity, and elevate the cytotoxicity of natural killer cells against YAC-1 lymphoma cells. Further validation in an immunosuppressed mouse model demonstrated that purified longan polysaccharide LP3 markedly restored concanavalin A-induced splenocyte proliferation, macrophage phagocytosis and natural killer cell cytotoxicity, while also promoting antibody production (Yi et al., 2011a; Yi et al., 2011b). This review systematically summarizes the structure-activity relationships of LP in terms of their immunomodulatory effects, noting that high-molecular-weight metabolites are key to their immunomodulatory activity and generally demonstrate superior efficacy in enhancing macrophage phagocytic activity (Yue et al., 2022). Both fresh and dried LP (LPX3 and LPG2) promote the proliferation of mesenteric lymph node cells and macrophages and enhance the phagocytic capacity of macrophages (Wang et al., 2024). High-molecular-weight water-soluble polysaccharide fractions from longan significantly enhance macrophage phagocytic activity (70%–97%) and NO production (Lan et al., 2021c). LP also promote the secretion of IL-6, IL-10, and IL-12 by dendritic cells, creating a microenvironment with a balance between pro-inflammatory and anti-inflammatory factors, and driving the differentiation of T cells into Th1 and Treg subsets (Wang et al., 2025). These effects directly enhance the body’s ability to phagocytose, process, and clear antigens, forming a line of defense in the immune system and regulating the immune system by activating immune cells.

Longan extract modulates immune system activity through multiple signaling pathways. LP initiate intracellular signal transduction by binding to pattern recognition receptors (PRRs), with TLR2 and TLR4 being the primary targets. LP4 activates the downstream PI3K/AKt and MyD88/TRAF6 pathways via TLR2/4. Following receptor blockade with anti-TLR2 and/or anti-TLR4 antibodies, mRNA expression of AKT, PI3K, TRAF6, and MyD88 in macrophages was significantly suppressed, confirming that both pathways are dependent on TLR2/4 (Lan et al., 2021b). LPD2 induces macrophage activation via the MyD88/IRAK4-TRAF6 signaling pathway mediated by TLR2 and TLR4. Anti-TLR2 and anti-TLR4 monoclonal antibodies significantly inhibited LPD2-mediated production of NO and IL-6, as well as the mRNA expression of MyD88, IRAK4, TRAF6, and iNOS (Rong et al., 2019). LPG (Longan Polysaccharides) primarily activates macrophages through the TLR2/4-mediated TRAM/TRAF6 signaling pathway, while also engaging the CLR-mediated Src/Raf1 NF-κB pathway. RNA-seq analysis identified TNF and chemokine as key genes (Wang et al., 2024). LPIIa activates macrophages via TLR4 and TLR2, with downstream signaling dependent on the p38 MAPK and NF-κB pathways (Yi et al., 2015). LP activate dendritic cells (DCs) via the TLR-NF-κB pathway, upregulate the expression of CD80, CD86, and MHC class II, and drive T cell differentiation toward Th1 and Treg subsets (Wannavijit et al., 2025). Although the upstream signaling pathways of different longan polysaccharide fractions all rely on TLR2 and TLR4, the selection of downstream adaptor proteins (such as MyD88, TRAM, and IRAK4) varies, thereby determining the final direction of the immune response in macrophages or dendritic cells.

It is worth noting that there are distinct differences in the signaling mechanisms of dried and fresh longan: LPG (Longan polysaccharides) from dried longan primarily activate NF-κB via the TLR2/4-TRAM/TRAF6 and CLR-Src/Raf1 pathways; in contrast, LPX (Longan polysaccharides) from fresh longan activate MAPK and NF-κB via the CLR-Bcl10/MALT1 and NLR-Rip2/TAK1 pathways (Wang et al., 2024). Low-molecular-weight polysaccharides in longan can bind to MD2, elicit a macrophage response mediated by TLR1/2, and are regulated via Ca^2+^ signaling pathways. High-molecular-weight polysaccharides primarily activate macrophages via the TLR1/2 and TLR7/8 receptors (Lan et al., 2021a). Longan extract can also reduce NO production by inhibiting iNOS expression, IκBα degradation, and the activation of NF-κB and AP-1, demonstrating its bidirectional regulatory properties in anti-inflammatory processes (Kunworarath et al., 2016).

### 4.4 Anti-hyperglycemic activity

Hyperglycemia induces excessive oxidative stress, eNOS uncoupling, and NF-κB-mediated vascular inflammation by activating the polyol (Schleicher and Friess, 2007), AGE, PKC (Xiao et al., 2023), and hexosamine pathways (Hussain, 2025), directly leading to endothelial dysfunction and thereby becoming an independent driver of CVD such as atherosclerosis (Esper et al., 2008).

Recent key research findings indicate that the mechanism by which longan pulp extract lowers blood sugar is not limited to peripheral insulin sensitization but also involves appetite-regulating pathways in the central nervous system. Longan fruit extract (LE) not only significantly reduced fasting blood glucose levels and excessive epididymal fat accumulation, but also improved glucose tolerance and insulin sensitivity. Crucially, LE supplementation significantly reduced food intake in mice; this mechanism is closely associated with increased activity in POMC-producing neurons and decreased activity in agouti-related protein (AgRP)-expressing neurons in the arcuate nucleus of the hypothalamus (Eo et al., 2023).

A study identified nine phenolic metabolites in a longan peel polyphenol extract using HPLC-DAD/MS analysis, confirming that these metabolites reduce postprandial hyperglycemia by inhibiting α-glucosidase in the intestinal lumen and improve glucose tolerance in mice following short-term oral administration (Eo et al., 2023; He et al., 2021). More systematic research conducted over the past 5 years has involved the systematic quantification and bioactivity screening of polyphenols (phenolic acids and flavonoids) and alkaloids in longan peel and seeds. The findings indicate that the total polyphenol and total flavonoid content in longan peel is slightly higher than that in the seeds, while the alkaloids in the peel have been identified as having potential for development into antihyperglycemic drugs (Tang et al., 2019). In earlier mechanistic studies, hot-water extracts of longan peel have been shown *in vitro* to inhibit the activity of α-glucosidase and β-galactosidase, with gallic acid and ellagic acid identified as the primary phenolic inhibitory metabolites (Li et al., 2016).

Longan seeds are another abundant byproduct rich in polyphenols and flavonoids; longan seed polyphenols (LSPs) possess a variety of bioactive properties and account for 17% of the longan fruit (Zhao et al., 2019). LSPs significantly inhibited postprandial blood glucose elevation in mice in a dose-dependent manner. *In vitro* enzyme kinetic analysis further revealed that LSPs inhibit α-amylase activity through a mixed mechanism. LSPs primarily bind to α-amylase at a single site via hydrogen bonds and van der Waals forces, thereby altering the enzyme’s secondary structure. This modification results in a more compact main chain and a more flexible active ring, preventing substrates from reaching the enzyme’s active site and consequently reducing enzyme activity. This study clearly demonstrates that LSPs have application potential as hypoglycemic components in functional foods (He et al., 2021). A study by Tang et al. also demonstrated that the flavonoids found in seeds have the potential to be developed into antihyperglycemic drugs (Tang et al., 2019).

A recent study examined in depth the mechanisms underlying the anti-type 2 diabetes effects of longan leaf extract (DLC), which contains key bioactive metabolites such as quercetin, kaempferol, and quercetin glycosides. The study demonstrated that DLC intervention significantly alleviated pathological weight loss, reduced fasting blood glucose levels, restored glucose homeostasis, and improved dyslipidemia (Zheng et al., 2025). DLC restores gut microbial diversity, reverses T2DM-induced gut dysbiosis, and reshapes the SCFA profile by increasing butyrate, isobutyrate, and 2-methylbutyrate while decreasing acetate and propionate, an elevation of butyrate that is critical for intestinal barrier maintenance and insulin sensitivity improvement. Non-targeted metabolomics further demonstrated that DLC reprograms amino acid, carbohydrate, purine, and biotin metabolism, with specific gut bacterial genera significantly correlated with the altered fecal metabolites. These findings clearly indicate that DLC may exert its blood glucose-lowering effects by regulating gut microbiota composition, SCFA levels, and fecal metabolites (Zheng et al., 2025). Meanwhile, a study focusing on a 95% ethanol extract of longan leaves (LYY) demonstrated that by targeting the remodeling of the gut microbiota and increasing short-chain fatty acid (SCFA) production, it systemically alleviates insulin resistance and inhibits hepatic gluconeogenesis via the “microbiota-SCFAs-AMPK/Akt” axis. This further confirms that LYY exerts its anti-T2DM effects through the regulation of metabolic pathways and the gut microbiota (Lu et al., 2026). Longan leaves can regulate metabolic disorders caused by type 2 diabetes by modulating amino acid metabolism, amino acid synthesis, and the tricarboxylic acid cycle, thereby offering a new therapeutic approach for the clinical treatment of type 2 diabetes (Liang et al., 2025).

### 4.5 Regulate blood lipid levels

Hyperlipidemia is a key factor driving core cardiovascular pathological processes—such as atherosclerosis, myocardial ischemia, and endothelial dysfunction—by inducing excessive oxidative stress and chronic inflammation (Zhou et al., 2025). Different parts of the longan fruit are rich in polyphenols, flavonoids, alkaloids, and polysaccharides; its lipid-lowering effects are achieved through a multifaceted synergistic mechanism that involves inhibiting lipid absorption, regulating hepatic lipid metabolism, and modulating the gut microbiota (Hu et al., 2025; Zeng et al., 2024).

The absorption of dietary fat begins with the hydrolysis of triglycerides by pancreatic lipase; inhibition of this enzyme reduces the formation of free fatty acids and monoglycerides, thereby lowering postprandial blood lipid levels. *In vitro*, an extract of longan flower water significantly inhibits pancreatic lipase activity, with a half-maximal inhibitory concentration (IC50) of 0.313 mg/mL (Li et al., 2016). In a rat model fed a high-calorie diet, after 9 weeks of oral administration of a 2.5% longan flower water extract (LFWE), serum triglycerides (TG) and the atherosclerosis index both decreased significantly, accompanied by increased fecal triglyceride excretion and marked inhibition of pancreatic lipase activity (Yang et al., 2010). At the same time, longan flower extract exerts a synergistic lipid-lowering effect by inhibiting intestinal absorption (Yang et al., 2010). Therefore, an extract of longan flower water inhibits pancreatic lipase, increase the excretion of dietary fat, and suppress the digestion and absorption of lipids in the intestine.

The liver is the central hub of endogenous lipid homeostasis. Longan extract precisely regulates the balance between lipid synthesis and catabolism through a network of nuclear receptor transcription factors. This facilitates lipid catabolism and cholesterol clearance in the liver. SREBP-1c is a key transcription factor that regulates fatty acid and triglyceride synthesis. The mRNA expression of SREBP-1c and FAS in the livers of rats fed a high-calorie diet was significantly downregulated by LFWE (Yang et al., 2010). Longan seed polyphenols activate AMP-activated protein kinase (AMPK), which phosphorylates SREBP-1c and inhibits its nuclear translocation, thereby reducing the expression of target genes such as FAS and significantly alleviating hepatic steatosis (Tan et al., 2023). Peroxisome proliferator-activated receptor alpha (PPARα) is the primary nuclear receptor involved in fatty acid oxidation in the liver. Longan flower extract can upregulate PPARα gene expression in rat livers (Yang et al., 2010). LPPE prevents weight gain and reduces serum and hepatic triglycerides (TG) and total cholesterol (TC) in mice with high-fat diet-induced obesity. The molecular basis for this effect is a significant upregulation of PPARα and liver X receptor α (LXRα) protein expression, accompanied by increased expression of the downstream target genes CYP7A1 and CYP27A1 (Tan et al., 2023). PPARα activation promotes mitochondrial fatty acid β-oxidation and accelerates triglyceride breakdown. LXRα drives the conversion of cholesterol into bile acids. Longan LPPE upregulates LXRα and its target genes CYP7A1 and CYP27A1, promoting the excretion of cholesterol through the gut (Tan et al., 2023). In addition, longan flower extract can upregulate the expression of the liver low-density lipoprotein receptor (LDLR) gene, thereby enhancing the uptake and clearance of serum LDL-C (Yang et al., 2010).

Oxidative stress and low-grade inflammation exacerbate lipid metabolism disorders. Thanks to its potent free radical scavenging capacity and anti-inflammatory activity, longan extract indirectly supports the normal functioning of lipid metabolism pathways. In a rat model of non-alcoholic fatty liver disease, polyphenol-rich longan flower extract not only reduced serum and hepatic lipid levels but also significantly inhibited hepatic lipid peroxidation, restored the activity of superoxide dismutase (SOD) and glutathione peroxidase (GPx), and downregulated the expression of matrix metalloproteinases MMP-2 and MMP-9 (Yang et al., 2010). Increased MMP-2/9 activity is associated with the initiation of liver fibrosis; its inhibition suggests that the extract may block early events in the progression from fatty liver to fibrosis. Following administration of an ethanol extract of longan leaves to rats with type 2 diabetes, serum levels of the pro-inflammatory cytokines IL-6 and TNF-α decreased significantly, accompanied by improvements in lipid profiles (TC, TG, LDL-C) (Lu et al., 2026).

### 4.6 Regulate nitric oxide

NO is a key regulatory molecule in vascular endothelial function and plays an indispensable role in maintaining vascular homeostasis (Huang et al., 2012). The biological functions of NO are also profoundly influenced by the differentiation of its producing enzyme subtypes (constitutive eNOS/nNOS and inducible iNOS): at physiological levels, NO primarily maintains vascular protective functions, whereas the large amounts of NO continuously produced by iNOS under inflammatory conditions may promote endothelial damage and the progression of atherosclerosis through the generation of reactive nitrogen species such as peroxynitrite (Gliozzi et al., 2019; Li and Förstermann, 2000). Given the dual effects of NO—its concentration-dependent and spatiotemporal-specific properties—the search for natural resources that can naturally regulate endothelial function and NO homeostasis through dietary means has become a key focus in cardiovascular prevention research.

Extracts from various parts of the longan fruit are rich in active polyphenolic metabolites such as proanthocyanidins, corilagin, gallic acid, and ellagic acid. They exert a protective regulatory effect on vascular endothelial function through two mechanisms: directly activating endothelial NO release and inhibiting inflammation-driven iNOS/NO production. Notably, longan extracts exhibit a distinct bidirectional effect on NO synthesis regulation: under normal conditions, they promote the protective eNOS pathway, whereas under inflammatory conditions, they inhibit the destructive iNOS pathway (Kunworarath et al., 2016; Tang et al., 2019; Wattanapitayakul et al., 2021).

Researchers used the human endothelial cell line EA.hy926 to conduct an *in vitro* functional screening of 10 tropical fruits to assess their potential cardiovascular protective benefits. The results showed that longan effectively stimulates the release of NO in endothelial cells (Wattanapitayakul et al., 2021). The ability of longan to promote endothelial NO release is closely linked to its high content of antioxidant polyphenols. Research has shown that gallic acid promotes NO production in the vascular endothelium by activating eNOS phosphorylation, while corilagin has been shown to possess multiple activities, including tyrosinase inhibition, antioxidant effects, nitrite scavenging, and the blocking of nitrosamine synthesis (Tang et al., 2019; Zeng et al., 2024). Several known eNOS-activating metabolites in longan can synergistically promote endothelial NO production.

When macrophages are activated by pro-inflammatory stimuli such as LPS, iNOS expression is dramatically upregulated, continuously catalyzing the production of NO at concentrations ranging from nanomolar to micromolar levels. At these concentrations, NO no longer functions primarily as a physiological signaling molecule but reacts with superoxide anions to form the potent oxidizing agent peroxynitrite (ONOO^−^), which exacerbates tissue damage and vascular endothelial dysfunction through protein nitrosylation, lipid peroxidation, and DNA damage (Pacher et al., 2007). The efficacy of extracts from different parts of the longan fruit in inhibiting iNOS varies. One study systematically compared the anti-inflammatory activity of extracts from longan flowers, seeds, and pulp. In an LPS-stimulated RAW264.7 macrophage model, longan flower extract exhibited the strongest inhibitory effect on NO production (IC50 = 128.2 μg/mL), far surpassing that of seed extract (IC50 = 1,127.4 μg/mL) and pulp extract (IC50 = 1,260.2 μg/mL) (Kunworarath et al., 2016). The study further confirmed via Western blot that the inhibitory effect of longan flower extract on NO is attributed to its downregulation of iNOS protein expression, rather than direct inhibition of the catalytic activity of already expressed iNOS enzymes (Ho et al., 2007). Longan pericarp similarly exhibited inhibitory activity toward the iNOS/NO pathway, and the aqueous extract of longan pericarp, WLP, suppressed LPS-induced NO production in macrophages (Huang et al., 2012).

### 4.7 Angiotensin-converting enzyme (ACE) inhibitor

The renin-angiotensin system (RAS) plays a key role in the regulation of endothelial function. Angiotensin-converting enzyme (ACE) catalyzes the conversion of inactive angiotensin I into the potent vasoconstrictor angiotensin II, while simultaneously degrading bradykinin, which possesses vasodilatory activity (Igić and Škrbić, 2014).

Longan seeds are an important source of ACE-inhibitory peptides. Following sequential hydrolysis of longan seed protein with pepsin and trypsin, the ultrafiltration fraction (molecular weight <5 kDa) exhibited the strongest ACE-inhibitory activity, with an IC50 value of 0.43 ± 0.011 mg protein/mL. A study has successfully isolated and identified two novel ACE-inhibitory peptides, ETSGMKPTEL and ISSMGILVCL, from longan seeds. Both peptides inhibit ACE activity through a non-competitive mechanism, meaning that they do not directly compete with the substrate for the catalytic active site but instead bind to an allosteric site outside the substrate-binding pocket, thereby reducing the maximum reaction rate of the enzyme. These two peptides primarily interact with amino acid residues in the active pocket of ACE through hydrogen bonding, which stabilizes the conformation of the peptide-enzyme complex and results in the inhibition of enzymatic activity. The discovery of these two peptides provides important molecular-level evidence supporting the use of longan seed protein as a natural source of ACE-inhibitory peptides (Nuchprapha et al., 2020).

### 4.8 Mechanistic integration and pathway crosstalk

The mechanistic interplay between antioxidant, inflammatory, NO-related, and immune pathways is critical for understanding the full protective capacity of longan. Oxidative stress serves as an upstream driver of endothelial injury: hyperglycaemia, hyperlipidaemia, and advanced glycation end-products generate excessive ROS, which activate apoptosis signal-regulating kinase 1 (ASK1) and its downstream MKKs, leading to JNK/p38 MAPK activation and NF-κB nuclear translocation (Matsuzawa and Ichijo, 2005). Longan polyphenols such as corilagin and longan polysaccharides interrupt this cascade by directly scavenging radicals and inducing Nrf2/ARE-dependent antioxidant enzymes, thereby removing the initial trigger for inflammation (Bai et al., 2019; Wang et al., 2024). Nitric oxide occupies a central position that links metabolic stress with inflammatory responses. Physiological eNOS-derived NO exerts endogenous anti-inflammatory effects through S-nitrosylation of the NF-κB p65 subunit, which inhibits its DNA-binding activity (Reynaert et al., 2004). In contrast, iNOS-derived NO reacts with superoxide to form peroxynitrite, which depletes tetrahydrobiopterin and causes eNOS uncoupling, converting eNOS into a superoxide-producing enzyme (Förstermann and Sessa, 2012; Pacher et al., 2007). Longan extracts exhibit a unique dual regulatory advantage: they promote eNOS phosphorylation while suppressing iNOS transcription via NF-κB inhibition, thus restoring physiological NO balance and rescuing eNOS function (Kunworarath et al., 2016; Tang et al., 2019; Wattanapitayakul et al., 2021). Furthermore, transcellular crosstalk between immune cells and the endothelium amplifies the local inflammatory milieu. Chronic high-fat feeding disrupts gut microbiota and increases intestinal permeability, allowing LPS to enter the circulation and activate TLR4 on perivascular adipose tissue macrophages. These activated macrophages secrete TNF-α, which engages TNFR1 on endothelial cells and triggers NADPH oxidase-dependent ROS production, establishing a positive feedback loop of inflammation and dysfunction (Hajer et al., 2008). Longan polysaccharides interrupt this loop through TLR2/4-MyD88-mediated pathways, polarising macrophages toward an anti-inflammatory phenotype that reduces TNF-α and IL-6 secretion while promoting IL-10 release (Lan et al., 2021b; Rong et al., 2019). These pathways are not isolated; oxidative stress drives inflammation and disrupts NO balance, NO exerts context-dependent protective or detrimental effects, and immune activation generates paracrine signals that further compromise endothelial redox status. By simultaneously intervening at these interconnected points, longan-derived metabolites provide a comprehensive strategy for endothelial protection that outperforms single-target approaches.

## 5 Translational challenges

Despite the compelling preclinical evidence for the vascular protective effects of longan, several critical translational gaps currently impede the clinical application of longan-derived bioactive components. These obstacles encompass incomplete characterisation of oral bioavailability, insufficient safety and dosage data, undefined botanical drug interaction risks, and a striking absence of rigorous human trials that directly evaluate endothelial function. Addressing these barriers is essential to realise the full potential of longan as a functional food metabolite and nutraceutical agent for cardiovascular health.

### 5.1 Oral bioavailability and gastrointestinal fate

The oral bioavailability of longan bioactive constituents remains poorly characterized, which represents a fundamental barrier to clinical translation. For polyphenolic metabolites such as gallic acid, ellagic acid, and corilagin, the principal obstacles include poor aqueous solubility, extensive first pass metabolism, and rapid systemic clearance. Gallic acid undergoes extensive phase II conjugation in the gut and liver, and sulfated and glucuronidated metabolites predominate in circulation (Shahrzad et al., 2001). Ellagic acid exhibits exceptionally low bioavailability, which results from poor intestinal absorption and rapid conversion to urolithins by gut microbiota (Espín et al., 2013; Tang et al., 2019). Corilagin displays an absolute oral bioavailability of only 10.7% in rats, and its Cmax of 87.64 ng/mL is far below the *in vitro* effective concentrations that range from 1 to 200 μg/mL (Wang et al., 2026; Zheng et al., 2016). For high molecular weight polysaccharides that exceed 100 kDa, the gastrointestinal barrier prevents systemic absorption. These macromolecules resist digestion and are not absorbed intact, and their biological effects are likely mediated through local interactions with TLR2 and TLR4 on intestinal immune cells (Lan et al., 2021c; Lin et al., 2015; Yue et al., 2022; Zhao et al., 2025). The gut microbiota exerts a dual role, as it ferments polysaccharides into short chain fatty acids and metabolises polyphenols into bioactive products that possess altered pharmacokinetic profiles. These quantitative data reveal a fundamental disconnect between *in vitro* effective concentrations and achievable systemic levels after oral administration. This gap may be partly reconciled by local gastrointestinal effects, gut microbial metabolism, tissue accumulation during chronic exposure, or synergistic interactions among constituents. Systematic pharmacokinetic studies that characterize the ADME profiles of individual constituents and their metabolites are urgently needed, and such data will inform delivery strategies that include nanoparticle encapsulation and are designed to enhance bioavailability.

### 5.2 Safety profile and dosage considerations

The safety profile of longan extracts lacks systematic evaluation, and well-defined dosage regimens have not been established. Longan is safe as food, but concentrated extracts at pharmacological doses remain poorly characterized. Preclinical studies show tolerance at 100–400 mg/kg in rodents without mortality or organ toxicity. However, the NOAEL has not been determined, and chronic toxicity data are absent. Dose-response relationships for endothelial function and related biomarkers have not been established. Optimal dosing strategies remain empirical. Future research should prioritise chronic toxicity studies and dose-ranging trials that incorporate validated biomarkers to define the therapeutic window.

### 5.3 Botanical drug interaction risks

The potential for botanical drug interactions with longan extracts remains uninvestigated. Many plant-derived polyphenols and polysaccharides modulate CYP enzymes and drug transporters. Gallic acid and ellagic acid inhibit CYP3A4, CYP2D6, and CYP2C9 *in vitro*, suggesting possible interactions with cardiovascular drugs (Duda-Madej et al., 2025; Vijayakumar et al., 2015). Longan polysaccharides may affect P-glycoprotein-mediated efflux, though this is unconfirmed. Patients using longan-based nutraceuticals are often on multiple cardiovascular medications. The lack of interaction data is a critical knowledge gap that requires targeted *in vitro* and clinical pharmacokinetic studies before clinical recommendations can be made.

## 6 Methodological quality and evidence hierarchy of the reviewed studies

To critically appraise the pharmacological evidence for longan’s vascular effects, we evaluated each included study against standardized methodological criteria that encompass extraction characterization, model selection, dose ranging, control usage, and reporting quality. Details of this appraisal across the twelve assessment domains are presented in Table 4.

**TABLE 4:** Methodological quality assessment of the included studies.

Assessment domain	Criterion for adequate quality	Current evidence profile	Key limitations	Rating
Taxonomic authentication	Voucher specimen deposited in recognized herbarium	Minority provided voucher numbers	Chemotypic variation risk uncontrolled	Moderate
Extract characterization	Marker quantification (HPLC/UPLC-MS/MS) with batch consistency	Majority used crude extracts; some quantified phenolics	Yield, solvent, temperature, and batch data often omitted	Moderate
Model selection and translational relevance	Multi-model (*in vitro* + *in vivo* + human)	∼70–80% *in vitro*; fewer rodents; no human RCTs	Heavy reliance on cell-based assays	Moderate
Pathophysiological relevance	Models recapitulating chronic vascular disease	LPS-stimulated acute endotoxemia dominates	Poor representation of chronic atherosclerosis	Low
Cellular model appropriateness	Primary cells or *in vivo* endothelium	Immortalized cell lines commonly used	Potential signaling alterations vs. primary cells	Moderate
Dose–response characterization	Multiple doses with minimum effective concentration	*In vitro*: 1–200 μg/mL; *in vivo*: 100–400 mg/kg	Relevance to human systemic exposure unclear	Adequate
In vitro–in vivo dose bridging	Effective *in vitro* concentrations achievable systemically	Relationship unexplored	Oral bioavailability and plasma levels unknown	Low
Route of administration	Oral, mimicking human dietary exposure	Gavage or intraperitoneal injection common	Bypasses first-pass metabolism	Low
Intervention duration	Chronic exposure relevant to dietary habits	Typically 1–4 weeks	Insufficient for long-term dietary modeling	Moderate
Positive controls	Reference comparator drugs included	<40% included positive controls	Potency benchmarking limited	Moderate
Human clinical evidence	RCT with validated endothelial endpoints	None available	No validated biomarker data in humans	Very low

Overall rating: Moderate. core mechanistic conclusions are supported by replicated preclinical findings; however, translational inference is constrained by predominant *in vitro* designs, inconsistent extract characterization, and the absence of human interventional trials.

A major and pervasive weakness across the evidence base is inconsistent test material characterization. While some studies employed HPLC or UPLC tandem MS to quantify phenolic markers such as gallic acid, corilagin, and ellagic acid, many others used crude extracts without specifying yield, solvent composition, temperature, or batch variability. This deficiency compromises comparability between studies and precludes definitive attribution of observed effects to specific constituents.

The evidence base is dominated by *in vitro* models, which account for approximately 70–80 percent of included studies, with fewer rodent investigations and no human interventional trials. Cell free antioxidant assays provide chemical reactivity data but ignore bioavailability and cellular metabolism, whereas LPS stimulated macrophage models assess acute endotoxemia rather than the chronic low grade inflammation that characterizes atherosclerosis. *In vitro* concentrations span 1–200 μg per milliliter, yet their relationship to achievable systemic concentrations after oral administration remains unexplored given the poor bioavailability of polyphenols. In addition, less than 40 percent of studies included positive comparator drugs, which limits benchmarking of relative potency.

Based on this assessment, we assigned hierarchical weight to the findings. Highest weight was accorded to effects replicated across multiple experimental models with consistent dose response relationships and appropriate positive controls. Moderate weight was assigned to rigorous single model studies that used characterized extracts. Lowest weight was given to investigations that lacked extract characterization, omitted positive controls, or provided no dose response data. This weighting strategy informed the interpretative emphasis applied throughout the review.

Overall, the methodological quality of the included studies is variable. It is moderate for mechanistic plausibility but low for clinical translational inference. The predominance of *in vitro* models, inadequate extract characterization in many reports, absence of positive comparator drugs, and the complete lack of human randomized controlled trials constrain the strength of clinical recommendations. Nevertheless, high weight evidence, defined as findings replicated across independent models with consistent dose response patterns, robustly supports the core mechanistic conclusions, particularly the bidirectional regulation of nitric oxide homeostasis, Nrf2 mediated antioxidant defense, and nuclear factor kappa B inhibition. Conversely, evidence derived from single model studies using crude extracts warrants cautious interpretation. These methodological limitations directly inform the translational barriers detailed in Section 5 and the research priorities outlined in Section 7.

## 7 Future directions for clinical translation

To address the translational barriers identified above, future research should prioritize three directions: component-resolved mechanistic studies, comprehensive pharmacokinetic and safety evaluations, and well-designed randomized controlled trials. These directions are elaborated in the following subsections.

### 7.1 Traditional culinary and nutraceutical applications

Longan has been consumed as both a food and a medicinal agent for centuries across Asia, and its dual-use status provides a foundation for nutraceutical development. In traditional Chinese medicine, longan aril is classified as a tonic botanical drug that tonifies the heart and spleen, nourishes the blood, and calms the mind (Zhang et al., 2025; Zhao et al., 2025). It is incorporated into more than sixty traditional Chinese patent medicines, including Guipi Pills, Renshen Yangrong Pills, and Anshen Yangxin Pills, which are used to treat palpitations, insomnia, forgetfulness, and anxiety (Ma et al., 2009; Park et al., 2010). As a food-medicine homologue, longan is widely used in health foods and culinary preparations such as dried longan, longan wine, and longan-red date porridge. The market for longan-based functional foods is expanding rapidly, yet current commercial products lack standardized bioactive content and rigorous scientific substantiation of their health claims. The establishment of standardized extraction protocols, quality control markers, and validated bioactivity assays is essential to translate traditional use into evidence-based functional food products with verifiable health benefits.

Beyond these standardization needs, the mechanistic evidence presented in this review carries direct translational implications for specific cardiovascular conditions. For endothelial dysfunction, longan polyphenols enhance eNOS/NO bioavailability and suppress oxidative stress and inflammation, supporting their use for preserving endothelial integrity in at-risk populations. For hypertension, ACE-inhibitory peptides from seeds (Nuchprapha et al., 2020) and eNOS-mediated vasorelaxation position seed extracts for mild-to-moderate hypertension. For atherosclerosis, antioxidant, anti-inflammatory, immunomodulatory, and lipid-regulating actions collectively inhibit plaque progression (Lan et al., 2021b; Wang et al., 2024; Yang et al., 2010; Tan et al., 2023). For diabetic complications, hypoglycaemic effects of polyphenols (He et al., 2021; Zheng et al., 2025; Eo et al., 2023) address hyperglycaemic injury, while AGE suppression may slow microvascular damage. These findings support a three-tier framework: whole fruit for general wellness; standardized extracts for targeted nutraceuticals; and purified metabolites for pharmaceutical development.

### 7.2 Clinical evidence and translational gaps

The most critical translational gap is the complete absence of randomised controlled trials that evaluate the effects of longan extracts on endothelial function or cardiovascular outcomes in humans. To date, clinical investigations have been limited to a small number of observational studies and uncontrolled interventions with small sample sizes. One trial found subjective sleep improvements in elderly individuals with insomnia after longan juice consumption, without objective polysomnographic confirmation (Poon et al., 2012). No study has measured flow-mediated dilation, circulating endothelial microparticles, or other validated biomarkers of endothelial function in response to longan intervention. The absence of dose-finding studies, pharmacokinetic-pharmacodynamic modelling, and biomarker-based efficacy assessments means that the optimal formulation, dose, and patient population remain undefined. Despite these limitations, the extensive preclinical evidence provides a strong mechanistic rationale for conducting rigorous human trials. These limitations collectively underscore the urgent need for rigorous clinical trials to bridge the gap between preclinical promise and clinical application.

### 7.3 Future perspectives and research priorities

To translate mechanistic evidence into clinical strategies, three research directions should be prioritized. First, component-resolved mechanistic studies with purified metabolites, including gallic acid, ellagic acid, corilagin, ACE-inhibitory peptides, and high-molecular-weight polysaccharides, are needed to identify the most active entities and their target interactions. Beyond single-metabolite profiling, systematic combination studies using factorial dose-matrix designs and isobologram analysis should be undertaken to quantitatively assess whether the combined effects of these constituents are truly synergistic, additive, or simply independent—such studies are essential to substantiate the widely invoked but mechanistically unproven concept of ‘multi-target synergy’ in longan pharmacology. Second, comprehensive pharmacokinetic and safety evaluations under standardized protocols should characterize ADME profiles, determine the NOAEL, and assess botanical drug interactions with cardiovascular drugs. Third, well-designed, placebo-controlled RCTs should assess standardized longan extracts in populations with early-stage endothelial dysfunction, using validated biomarkers such as FMD and circulating microparticles as endpoints. These three directions will provide the evidence base for developing longan-based nutraceuticals and pharmaceuticals.

## 8 Conclusion

The vascular protective effects of longan are not attributable to a single metabolite or a solitary pathway. Rather, they arise from the integrated actions of multiple bioactive constituents—including polyphenols, polysaccharides, and ACE-inhibitory peptides—that target distinct but interconnected pathological processes, namely oxidative stress, inflammation, immune dysregulation, metabolic imbalance, and impaired vascular tone. The synergistic interplay among these constituents and their targets differentiates longan from single-agent interventions and highlights its value as a multi-target dietary strategy for vascular protection.

The most critical mechanism underlying its vascular endothelial protection is the bidirectional regulation of NO homeostasis. Longan polyphenols promote protective eNOS-derived NO production under physiological conditions while inhibiting iNOS-driven excessive NO generation during inflammation. This NO-modulating action is complemented by potent suppression of the TNF-α/NF-κB and MAPK inflammatory cascades, direct radical scavenging, and upregulation of endogenous antioxidant defenses. These pathways do not operate in isolation. Oxidative stress fuels inflammatory activation, inflammation impairs NO bioavailability through eNOS uncoupling, and immune activation amplifies both oxidative and inflammatory injury, establishing a self-reinforcing pathological cycle. Longan-derived metabolites interrupt this cycle at multiple nodes, converting a pathological feedback loop into a coordinated defensive network. This network-based mechanism, together with systemic metabolic benefits including improved glucose tolerance, reduced lipogenesis, and ACE inhibition, collectively alleviates endothelial injury and maintains vascular homeostasis.

## Glossary

ABTS: 2,2′-azino-bis(3-ethylbenzothiazoline-6-sulfonic acid)
AP-1: Activator Protein 1
Ara: Arabinose
CVD: Cardiovascular disease
CAT: Catalase
DPPH: Diphenyl-picrylhydrazyl
FLP: The purified neutral polysaccharide from fresh longan
GalA: Galacturonic Acid
Gal: Galactose
Glc: Glucose
GSH-Px: Glutathione peroxidase
HPLC-DAD: High Performance Liquid Chromatography - Diode Array Detector
LFWE: Longan flower water extract
LP: Longan Polysaccharides
LP-E: Polysaccharides obtained by Enzyme treatment
LP-H: Polysaccharides obtained by the Hot-Water Extraction method
LP-S: Polysaccharides obtained by the superfine grinding method
LP-SE: Polysaccharides obtained by superfine grinding-assisted enzymatic treatment
LP-UE: Polysaccharide consists of arabinose, glucose and galactose linked through glycosidic bonds
LP1: A new water-soluble polysaccharide from longan pulp
LP4: A type of immunologically active polysaccharide that promotes cell proliferation and macrophage activity
LPD2: An active polysaccharide
LPG: Dried longan polysaccharide
LPPE: Longan by-product polyphenol extract
LPS: Lipopolysaccharide
LPS1: A water-soluble pure polysaccharide derived from longan pulp
LPX: Fresh longan polysaccharide
LSPs: Longan seed polyphenols
LWP: A longan fruit wine polysaccharide
MAPK: Mitogen-Activated Protein Kinase
MDA: Malondialdehyde
NF-κB: Nuclear Factor kappa-light-chain enhancer of activated B cells
NO: Nitric Oxide
TNF-α: Tumor Necrosis Factor-alpha
TG: Triglycerides
TC: Total cholesterol
IL-6: Interleukin-6
IL-1β: Interleukin-1β
**PGE**_**2**_: Prostaglandin E2
SOD: Superoxide dismutase
UPLC-MS/MS: Ultra Performance Liquid Chromatography-Tandem Mass Spectrometry
WLP: Water extracts from fruit peels
