# Supplementary material for: Vascular endothelial protective effects of longan (Dimocarpus longan Lour.) extract and underlying molecular mechanisms
Source: Front Pharmacol. 2026 Sep 3;17:1934125. doi: 10.3389/fphar.2026.1934125 (PMC13581752; doi:10.3389/fphar.2026.1934125)
Supplement: Supplementary file 1 [file DataSheet1.docx]

Supplementary Table 1

Sugar compounds of longan

| Number | Compounds | Molecular formulae | Molecular Structure Diagram | References |
| --- | --- | --- | --- | --- |
| 1 | L-Arabinose | C_5_H_10_O_5_ |  | (Yi et al., 2012) |
| 2 | Ribose | C_5_H_10_O_5_ |  | (Yi et al., 2012) |
| 3 | Xylose | C_5_H_10_O_5_ |  | (Yi et al., 2012) |
| 4 | dl-Galactose | C_6_H_12_O_6_ |  | (Yi et al., 2012) |
| 5 | Galacturonic acid | C_6_H_10_O_7_ |  | (Yi et al., 2012) |
| 6 | Rhamnose | C_6_H_12_O_5_ |  | (Yi et al., 2012) |
| 7 | Mannose | C_6_H_12_O_6_ |  | (Zhao et al., 2025) |
| 8 | Glucose | C_6_H_12_O_6_ |  | (Zhao et al., 2025) |
| 9 | Fructose | C_6_H_12_O_6_ |  | (Zhao et al., 2025) |
| 10 | Sucrose | C_12_H_22_O_11_ |  | (Zhao et al., 2025) |
| 11 | α-D-Glucose | C_6_H_12_O_6_ |  | (Zhao et al., 2025) |
| 12 | β-D-Glucose | C_6_H_12_O_6_ |  | (Zhao et al., 2025) |
