# Supplementary material for: Vascular endothelial protective effects of longan (Dimocarpus longan Lour.) extract and underlying molecular mechanisms
Source: Front Pharmacol. 2026 Sep 3;17:1934125. doi: 10.3389/fphar.2026.1934125 (PMC13581752; doi:10.3389/fphar.2026.1934125)
Supplement: Supplementary file 2 [file DataSheet2.docx]

Supplementary Table 2

Longan polysaccharide components

| Number | Name | Extraction Method | Mw (kDa) | Monosaccharid components(molar ratio) | Structure | Pharmacological Properties | Reference |
| --- | --- | --- | --- | --- | --- | --- | --- |
| 1 | LP1 | Hot water | 1.1 ×10^5^ | Glc: GalA: Ara: Gal = 5.39:1.04:0.74:0.21. | →4)-α-D-Glcp-(1→, →4,6)-β-D-Glcp-(1→, →2)-β-D-Fruf-(1→, and →2)-L-sorbose-(1→ | immunomodulatory activity. | (Meng et al., 2014) |
| 2 | LPS1 | Hot water | 108 |  | →6)- d - glc -(1→ | Immunological Activity and Antitumor Activity | (Zhu et al., 2013) |
| 3 | LPD2 | Hot water | 9.64 × 10^6^ | Ara:Man:Glc:Gal = 0.25:0.49:1:0.5 | （1→4）-β-Glc （1→6）-β-Man | immunomodulatory activity. | (Rong et al., 2019) |
| 4 | LP4 |  | 6.31 × 10^4^ | Man：Glc：GlcA：Gal：Xyl：Ara：GalA：Fuc：Rha=36:31:10:7:4:3:2:2 | （1→6）-β-Man、（1→4）-β-Glc和（1→6）-α-Glc | immunomodulatory activity. | (Lan, Li, et al., 2021) |
| 5 | LP-H | Hot water | 3.18 × 10^5^ | Rha:Ara:Xyl:Man:Glc:Gal = 2.82:30.38:2.56:4.01:32.26:28.46 |  |  | (Bai et al., 2017) |
| 6 | LP-U | Ultra-high Pressure | 2.91 × 10^5^ | Rha:Arab:Xyl:Man:Glc:Gal = 3.14:31.29:3.25:2.67:26.63:33.01 |  |  | (Bai et al., 2017) |
| 7 | LP-E | Enzyme treatment | 1.32 × 10^5^ | Rha:Ara:Xyl:Man:Glc:Gal = 5.11:40.21:0.87:5.22:19.28:29.31 |  |  | (Bai et al., 2017) |
| 8 | LP-UE | Ultra-high pressure assisted enzyme treatment | 1.19 × 10^5^ | Rha:Ara:Xyl:Man:Glc:Gal = 5.35:37.52:0.92:2.16:29.87:24.54 |  |  | (Bai et al., 2017) |
| 9 | LPIa | Hot water | 147 | Rha:Rib:Ara:Xyl:Man:Glc:Gal = 0.99:1.37:34.61:1.48:1.73:5.86:55.16 | →3)-α-Araf-(1→, →3,6)-β-Galp-(1→, α-Araf-(1→ and →5)- α-Araf-(1→ | Protect the gastrointestinal tract | (Bai et al., 2020) |
| 10 | LPIIa | Hot water | 159.3 | Rha:Rib:Ara:Xyl:Glc:Gal = 1.05:1:22.88:1.01:2.59:34.58 | →3)-α-Araf-(1→, →3,6)-β-Galp-(1→, α-Araf-(1→ and →5)- α-Araf-(1→ | Protect the gastrointestinal tract | (Bai et al., 2020) |
| 11 | LPIIIa | Hot water | 19.4 | Rha:Rib:Fuc:Ara:Man:Glc:Gal = 14.46:1.85:2.31:46.17:1.00:1.97:20.99 | →3)-α-Araf-(1→, →3,6)-β-Galp-(1→, →3)-β-GalpA-(1→, and α-Rhap-(l→ | Protect the gastrointestinal tract | (Bai et al., 2020) |
| 12 | LPIVa | Hot water | 44 | Rha:Rib:Ara:Man:Glc:Gal = 4.71:0.38:25.03:1.00:2.53:15.50 | →3)-α-Araf-(1→, →3,6)-β-Galp-(1→, →3)-β-GalpA-(1→, and α-Rhap-(l→ | Protect the gastrointestinal tract | (Bai et al., 2020) |
| 13 | LPsx | Hot water | 4102 | Glc:Ara:Aal:Man:Xyl = 95.9:1.0:0.6:0.4 | →6)-α-D-Glcp-(1→, →6)-β-D-Glcp-(1→ | immunomodulatory activity. | (Lan, Cheng, et al., 2021) |
| 14 | LP-F | Hot water | 109.62 | Rha: Ara: Xyl: Man: Glc: Gal = 9.29:48.55:1.83:3.28:14.33:22.72 |  |  | (Huang, Hong, Zhang, Dong, et al., 2019) |
| 15 | LP-0 | Hot water | 221.63 ± 2.41 |  |  | prebiotic activity | (Huang, Hong, Zhang, Dong, et al., 2019) |
| 16 | LP-6 | Hot water | 152.98 ± 1.06 |  |  | prebiotic activity | (Huang, Hong, Zhang, Dong, et al., 2019) |
| 17 | LP-12 | Hot water | 108.71 ± 5.55 |  |  | prebiotic activity | (Huang, Hong, Zhang, Dong, et al., 2019) |
| 18 | lp - 24 | Hot water | 109.62 ± 10.66 |  |  | prebiotic activity | (Huang, Hong, Zhang, Dong, et al., 2019) |
| 19 | LP-48 | Hot water | 136.83 ± 1.56 |  |  | prebiotic activity | (Huang, Hong, Zhang, Dong, et al., 2019) |
| 20 | LP-60 | Hot water | 139.42 ± 2.31 |  |  | prebiotic activity | (Huang, Hong, Zhang, Dong, et al., 2019) |
| 21 | LP-72 | Hot water | 152.31 ± 6.37 |  |  | prebiotic activity | (Huang, Hong, Zhang, Dong, et al., 2019) |
| 22 | FLP | Hot water | 1.31 × 10^4^ | Man:Glc = 0.59:1 | →6)-α-D-Glcp-(1→ | immunomodulatory activity. | (Gan et al., 2021) |
| 23 | DLP | Hot water | 1.06 × 10^4^ | Rha:Man:Glc:Gal = 0.2:0.21:1:0.2 | →6)-α-D- Glcp-(1→, →3)-β-D-Glcp-(1→, →3)-β-D-Galp-(1 → and → 3)-α-L-Rhap-(1→ | immunomodulatory activity. | (Gan et al., 2021) |
