# Supplementary material for: Vascular endothelial protective effects of longan (Dimocarpus longan Lour.) extract and underlying molecular mechanisms
Source: Front Pharmacol. 2026 Sep 3;17:1934125. doi: 10.3389/fphar.2026.1934125 (PMC13581752; doi:10.3389/fphar.2026.1934125)
Supplement: Supplementary file 3 [file DataSheet3.docx]

Supplementary Table 3

Longan polyphenol extract

| Number | Name | Molecular formula | Molecular Structure Diagram | References |
| --- | --- | --- | --- | --- |
| 1 | Phthalic acid | C_8_H_6_O_4_ |  | (Wang et al., 2020) |
| 2 | Gallic acid | C_7_H_6_O_5_ |  | (Zhao et al., 2025) |
| 3 | 4-O-Methylgallic acid | C_8_H_8_O_5_ |  | (Sun et al., 2007) |
| 4 | Brevifolin | C_12_H_8_O_6_ |  | (Zheng et al., 2009) |
| 5 | (±)-Naringenin | C_15_H_12_O_5_ |  | (Wang et al., 2020) |
| 6 | trans-Caffeic acid | C_9_H_8_O_4_ |  | (Zhao et al., 2025) |
| 7 | Syringic acid | C_9_H_10_O_5_ |  | (Khan et al., 2018) |
| 8 | Vanillic acid | C_8_H_8_O_4_ |  | (Khan et al., 2018) |
| 9 | trans-4- Hydroxycinnamic acid | C_9_H_8_O_3_ |  | (Khan et al., 2018) |
| 10 | Ellagic acid | C_14_H_6_O_8_ |  | (Rangkadilok et al., 2005) |
| 11 | Epicatechin | C_15_H_14_O_6_ |  | (Sun et al., 2007) |
| 12 | 4-Methylcatechol | C_7_H_8_O_2_ |  | (Khan et al., 2018) |
| 13 | Gallic glucoside | C_13_H_16_O_10_ |  | (Soong & Barlow, 2004) |
| 14 | Chlorogenic acid | C_16_H_18_O_9_ |  | (Zhao et al., 2025) |
| 15 | trans-Ferulic acid | C_10_H_10_O_4_ |  | (Zhao et al., 2025) |
| 16 | Quercetin 3-glucoside | C_21_H_20_O_12_ |  | (Khan et al., 2018) |
| 17 | Naringin | C_27_H_32_O_14_ |  | (Wang et al., 2020) |
| 18 | Corilagin | C_27_H_22_O_18_ |  | (Zhao et al., 2025) |
| 19 | Hesperidin | C_28_H_34_O_15_ |  | (Wang et al., 2020) |
| 20 | Methylhesperidin | C_29_H_36_O_15_ |  | (Wang et al., 2020) |
